# The functional activity of olfactory neural circuits in populations with subjective cognitive decline during olfactory tasks predicted by resting‐state functional magnetic resonance imaging

**DOI:** 10.1002/alz70856_101785

**Published:** 2025-12-25

**Authors:** Yajing Zhu, Bing Zhang

**Affiliations:** ^1^ Nanjing Drum Tower Hospital, Affiliated Hospital Clinical College of Nanjing Medical University, Nanjing, Jiangsu, China

## Abstract

**Background:**

Olfactory dysfunction is one of the earliest clinical symptoms in Alzheimer's Disease (AD) patients and is closely associated with cognitive decline. Subjective cognitive decline (SCD) is considered a risk group in the early stages of AD. Due to the difficulty in acquiring olfactory task‐based functional magnetic resonance imaging (fMRI) data, this study uses an end‐to‐end deep learning model to predict brain activity during olfactory tasks in the SCD population based on resting‐state fMRI (rs‐fMRI), in order to better understand early olfactory function changes in AD and the underlying neural mechanisms.

**Method:**

A total of 256 participants with subjective cognitive decline underwent rs‐fMRI and olfactory task‐based fMRI. Using an end‐to‐end deep learning model to predict the functional activity of the olfactory neural circuits in participants under odor stimulation based on rs‐fMRI. The prediction accuracy is calculated using the Pearson correlation coefficient.

**Result:**

Both individual prediction accuracy (*r* = 0.879, *p* < 0.001) and group prediction accuracy (*r* = 0.470, *p* =  0.02) showed a significant correlation between the predicted activation z‐scores and the actual activation z‐scores for POC.

**Conclusion:**

Regions in the SCD population under odor stimulation, providing an effective approach to exploring the neural mechanisms underlying olfactory function performance in large‐sample rs‐fMRI data.

